# The role of the general dental practitioner in the management of the hypodontia patient

**DOI:** 10.1038/s41415-023-6326-3

**Published:** 2023-10-13

**Authors:** Shivani Rana, Courtney Orloff, Deborah I. Bomfim, Martin P. Ashley, G. Steven Bassi

**Affiliations:** 41415431728001 Royal National ENT and Eastman Dental Hospital, London, WC1E 6DG, UK; 41415431728002 University Dental Hospital of Manchester, Manchester, M15 6FH, UK

## Abstract

The general dental practitioner (GDP) is usually the first person to suspect that a young patient is affected by hypodontia. The condition occurs rarely in the primary dentition but is relatively common in the permanent dentition. Between the ages of 7 and 12 years, failure of a permanent tooth to erupt as expected will lead the GDP to initiate and then contribute to the ideal management of the patient's condition. This ranges from reassurance and preventive measures to providing aspects of treatment in a long-term management plan, alongside a multidisciplinary specialist team and thereafter, delivery of life-long dental care.

## Key points

- To describe the role of the general dental practitioner for a patient who is affected by hypodontia.
- To understand the requirements for the general dental practitioner to support the hypodontia patient while they are being cared for by a multidisciplinary specialist team.
- To consider the potential for a future model of commissioned primary care dental services to contribute to hypodontia multidisciplinary teams.

## Introduction

A proportion of any population is affected by hypodontia,^1^ and it is therefore appropriate that those patients attending for dental care are managed by the dental team who can both recognise the condition and understand what treatment options exist.

The aim of this paper is to confirm the contributions of the general dental practitioner (GDP) and other members of the dental team in the management of a patient affected by hypodontia.

The GDP's role can be discussed under five separate but linked areas:

- Preservation of primary teeth
- Vigilance for suspected hypodontia
- Referral to secondary care services
- Delivery of aspects of the treatment plan
- Long-term monitoring.

## Preservation of primary teeth

As many patients affected by hypodontia will retain their primary teeth, a key role played by GDPs is in preservation of the retained teeth through preventive methods, in the form of oral hygiene instruction, dietary advice and fluoride provision, in line with the *Delivering better oral health toolkit*.^2^ The preservation of primary teeth is useful for functional and aesthetic reasons and also contributes to maintenance of space and alveolar bone, as this may be required for future interventions.^3^ The management of retained primary molar teeth is the focus of another paper in this issue by Patel *et al.*^4^

## Vigilance for suspected hypodontia between 7 and 12 years of age

GDPs have an important role in the early diagnosis of hypodontia. As part of regular clinical and radiographic assessments of children, the frequency of which should be tailored to individual caries risk and oral health needs,^5^ it is important that the GDP is aware of expected eruption dates and variations from the norm^6^ (Table 1).

**Table 1:** The average eruption period in years of age for lateral incisor, canine and premolar teeth, those that most commonly fail to develop and require treatment

Tooth	Eruption period (years of age)
Maxillary lateral incisor	8-9
Maxillary canine	11-12
Maxillary first premolar	10-11
Maxillary second premolar	10-12
Mandibular lateral incisor tooth	7-8
Mandibular canine	9-10
Mandibular first premolar	10-12
Mandibular second premolar	11-12

The GDP therefore needs to be vigilant and is responsible for assessing the developing dentition of each patient, especially through the period of 7-12 years. Clinically, hypodontia may be recognised when there is a delay of six months after eruption of the contralateral tooth in the same arch,^7^ or if six months has elapsed after exfoliation of the primary tooth, without evidence of eruption of its permanent successor.

There is also a significant association between hypodontia of the second premolar teeth and infraocclusion of primary molars, diminutive upper lateral incisors, enamel hypoplasia and impaction of the canines.^8^ These associations can often be of use to clinicians, as they may become apparent before the expected eruption date of permanent premolar teeth.

Hypodontia also commonly occurs in patients born with a cleft lip and palate and other craniofacial conditions. These patients are likely to already be under the care of specialist dental services but will need continuing care delivered by their GDP.

## Referral to secondary care services

Although orthodontic and restorative dentistry treatments are usually only provided once the permanent dentition is established, many patients with hypodontia will benefit from earlier referral. This will enable confirmation of the diagnosis, initial treatment planning, management of the primary teeth and, if required, interceptive orthodontics.

**Fig. 1:**
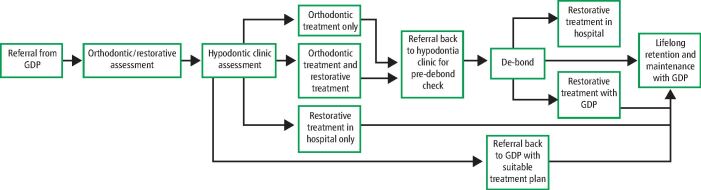
A care pathway model for hypodontia patients who receive shared care from both their GDP and a hypodontia MDT

The GDP has a responsibility to ensure the patient is referred to a suitably trained colleague who is able to assess the patient and, if necessary, direct the patient into the care of the multidisciplinary team (MDT) (Fig. 1). It is also the professional responsibility of all members of the dental team to communicate clearly with each other and with the patient throughout the shared care period.^9^

Management of hypodontia usually requires an MDT approach, with input from a paediatric dentist for those aged 15 or younger, and an orthodontist and restorative dentist at all stages.^10^^,^^11^ Additional involvement from an oral and maxillofacial surgeon may be required, depending on the extent of hypodontia, the presence of retained primary teeth and impacted permanent teeth, and for those patients requiring either orthognathic surgery or complex bone augmentation procedures. Occasionally, other specialties such as clinical psychology and speech and language therapy may become involved, depending on the individual patient's needs.

## Delivery of aspects of the treatment plan

As part of the shared care approach, the GDP will continue to see the patient at regular intervals to provide all the patient's routine dental care needs. This may include planned extraction of primary or permanent teeth in line with the orthodontic treatment plan. On completion of the specialist aspects of dental treatment, patients are generally discharged to the care of their GDP, potentially to assist in delivery of aspects of the treatment plan, which could include providing restorations for spaces and adjacent teeth and maintenance of the other specialist treatment received.

Ideally, each hypodontia patient would receive treatment for their dental needs from a local MDT of specialist colleagues with the training and capacity to deliver this. Due to a number of factors, this is not always possible. For example, in the UK, hypodontia MDTs often exist within dental teaching hospitals based in larger cities, and elsewhere, in district general hospitals.^12^ Some MDT services may not be able to deliver restorations to replace the missing teeth, such as resin-bonded bridges or dental implants, after the orthodontic treatment is completed. In addition, patients may not be able to travel large distances to receive the proposed treatment.

Rather than being disadvantaged by these service limitations, patients with hypodontia would therefore benefit from access to primary dental care services from one of the dental practices within a regional network, who have the capacity and clinical ability to deliver the treatment required. These practices become a key part of, rather than working alongside, the hypodontia MDT. Both these primary care GDPs and their supporting dental technician colleagues would have access to training, support and funding to ensure they have the competence and confidence to deliver these services. Similar regional network arrangements would benefit patients who require additional dental care while receiving specialist treatment for conditions such as cleft lip and palate, head and neck cancer and dental trauma.

## Long-term monitoring

GDPs also play a part in reviewing the condition of any restorations, such as resin-bonded bridges and dental implants provided, by periodic clinical and radiographic examination and by ensuring the patient is maintaining an adequate standard of dental hygiene. Recall appointments are also an opportunity to reinforce the importance of orthodontic retention and monitor for unwanted post-orthodontic treatment changes.^13^

Considering specialist treatment for hypodontia can take several years to complete, the patient may be under the care of a number of GDPs in the primary care setting, either because of staff changes within one practice or because the patient has moved to another practice during their period of treatment. Close communication of these changes and updates on progress of the treatment plan, shared between all those who contribute to the patient's dental care, is essential to ensure a good outcome is achieved.

## Conclusion

Patients affected by hypodontia are likely to require some dental intervention to preserve their primary teeth, to optimise the position of their permanent teeth and to replace their missing teeth. The patient must maintain regular visits to their GDP throughout any period of specialist dental care, as the specialist MDT members are not responsible for the routine aspects of dental care.

The impact of hypodontia and the treatment required to manage the condition are likely to have a life-long effect on the patient. The establishment and continuation of a long-term professional relationship with their GDP is as important to their dental health and to a successful outcome of treatment as the specialist treatment provided for their hypodontia condition.

Shared patient care between primary and secondary care services will both enhance the effective management of this condition and lead to improved patient outcomes for those that access services. Development of regional networks of dental practices to deliver aspects of care as part of the hypodontia MDT would reduce inequalities of access for patients.
