# Correction to: The role of the general dental practitioner in the management of the hypodontia patient

**DOI:** 10.1038/s41415-023-6545-7

**Published:** 2023-11-10

Author's correction note: 

General article *Br Dent J* 2023; **235:** 522-524.

The article 'The role of the general dental practitioner in the management of the hypodontia patient', written by Shivani Rana, Courtney Orloff, Deborah I. Bomfim, Martin P. Ashley and G. Steven Bassi, was originally published without Open Access. After publication in volume 235 issue 7, page 522-524 the authors decided to opt for Open Choice and to make the article an Open Access publication. Therefore, the copyright of the article has been changed to © The Author(s) 2023 and the article is forthwith distributed under the terms of the Creative Commons Attribution 4.0 International License, which permits use, sharing, adaptation, distribution and reproduction in any medium or format, as long as you give appropriate credit to the original author(s) and the source, provide a link to the Creative Commons licence, and indicate if changes were made. The images or other third party material in this article are included in the article's Creative Commons licence, unless indicated otherwise in a credit line to the material. If material is not included in the article's Creative Commons licence and your intended use is not permitted by statutory regulation or exceeds the permitted use, you will need to obtain permission directly from the copyright holder. To view a copy of this licence, visit . Open access funding enabled and organised by University of Manchester.

